# Hypoglycaemia and accident risk in people with type 2 diabetes mellitus treated with non-insulin antidiabetes drugs

**DOI:** 10.1111/dom.12031

**Published:** 2012-11-22

**Authors:** J E Signorovitch, D Macaulay, M Diener, Y Yan, E Q Wu, J-B Gruenberger, B M Frier

**Affiliations:** 1 Health Economics and Outcomes Research, Analysis Group, Inc.Boston, MA, USA; 2 Health Economics and Outcomes Research, Novartis Pharma AGBasel, Switzerland; 3 Department of Diabetes, Royal InfirmaryEdinburgh, UK

**Keywords:** antidiabetic drug, glycaemic control, type 2 diabetes

## Abstract

**Aims:**

To assess associations between hypoglycaemia and risk of accidents resulting in hospital visits among people with type 2 diabetes receiving antidiabetes drugs without insulin.

**Methods:**

People with type 2 diabetes who were not treated with insulin were identified from a US-based employer claims database (1998–2010). Following initiation of an antidiabetes drug, the occurrence of accidents resulting in hospital visits was compared between people with, and without, claims for hypoglycaemia using multivariable Cox proportional hazard models adjusted for demographics, comorbidities, prior treatments and prior medical service use. Additional analyses were stratified by age 65 years or older.

**Results:**

A total of N = 5582 people with claims for hypoglycaemia and N = 27 910 with no such claims were included. Accidents resulting in hospital visits occurred in 5.5 and 2.8% of people with, and without, hypoglycaemia, respectively. After adjusting for baseline characteristics, hypoglycaemia was associated with significantly increased hazards for any accident [hazard ratio (HR) 1.39, 95% CI 1.21–1.59, p < 0.001], accidental falls (HR 1.36, 95% CI 1.13–1.65, p < 0.001) and motor vehicle accidents (HR 1.82, 95% CI 1.18–2.80, p = 0.007). In age-stratified analyses, hypoglycaemia was associated with greater hazards of driving-related accidents in people younger than age 65 and falls in people aged 65 or older.

**Conclusions:**

In people with type 2 diabetes receiving antidiabetes drugs without insulin, hypoglycaemia was associated with a significantly higher risk of accidents resulting in hospital visits, including accidents related to driving and falls.

## Introduction

Good glycaemic control is a fundamental objective of the management of type 2 diabetes to minimize the risk of vascular complications of diabetes. However, strict glycaemic control and intensification of therapy can increase the risk of hypoglycaemia, especially for people treated with insulin or sulphonylureas [1,2]. Hypoglycaemia results in neuroglycopenia, which causes cognitive impairment and mood change and may progress to behavioural changes, reduced consciousness, seizure and coma [3,4]. These neurological impairments can, in turn, increase the risk of serious accidents and injuries, including those due to falls or motor vehicle accidents [4–7]. An appreciation of the associations between hypoglycaemia and accident risk is important for people with diabetes, physicians and policy makers [8–10].

Most evidence linking hypoglycaemia to increased accident risk is based on insulin-treated diabetes [4]. Automobile accidents have been associated with lower HbA1c in a largely insulin-treated population [11], and in people with type 1, but not type 2, diabetes [6]. Disrupted performance in simulated driving has been demonstrated during hypoglycaemia in people with type 1 diabetes [12,13]. An increased risk of falls, especially in the elderly, has been hypothesized for people experiencing hypoglycaemia. However, this association has been primarily confined to those treated with insulin [14,15]. An increased risk of fall-related fractures has been associated with hypoglycaemia in people with type 2 diabetes receiving oral antidiabetes drugs in Medicare, although not excluding use of insulin [16]. Because insulin treatment increases the frequency and severity of hypoglycaemia [17], associations with accidents in these studies may not generalize to populations with type 2 diabetes who are not treated with insulin.

Hypoglycaemia is often considered to be of limited consequence in people with non-insulin dependent diabetes. However, the hypoglycaemia risk in people receiving oral antidiabetes agents may be underestimated [1,17,18]. People with type 2 diabetes receiving sulphonylureas have reported hypoglycaemia in comparable proportions to those receiving insulin for less than 2 years, with 7% in both groups reporting severe hypoglycaemia and 39 and 51%, respectively, reporting mild hypoglycaemia [17]. The odds of hypoglycaemia among people receiving sulphonylureas are more than twice that among people receiving metformin [18]. Furthermore, from a public health perspective, the majority of people with type 2 diabetes are receiving oral antidiabetes drugs without insulin [19]. Associations between hypoglycaemia and accident risk in this population have not been well characterized and warrant further study.

The objective of this study was to assess the relationship between hypoglycaemia and risk of accidents resulting in hospital visits among people with type 2 diabetes receiving antidiabetes drugs without insulin. In addition, because associations between hypoglycaemia and accident risk may be modified by age [20,21], age-stratified analyses were conducted.

## Materials and Methods

Data were derived retrospectively from a large de-identified claims database covering more than 12 million employees, retirees, spouses and dependents from self-insured companies in the USA. The database includes enrolment data, medical service claims (classified as inpatient, emergency department or outpatient services), associated International Classification of Diseases, Ninth Revision, Clinical Modification (ICD-9-CM) diagnosis codes and prescription drug claims.

People with a recorded diagnosis of type 2 diabetes who had filled at least two prescriptions for an antidiabetes drug, but had no evidence of insulin use, were identified between 1 January 1998 and 31 March 2010. Type 2 diabetes was identified as the presence of ICD-9-CM code 250.xx (diabetes mellitus) without 250.x1 or 250.x3 (type 1 diabetes). Antidiabetes drugs were identified using generic product identifier codes. The first prescription fill for an antidiabetes drug was defined as the index date. Selected people were required to have continuous health plan enrolment for the year preceding the index date, which was defined as the baseline period. Selected people were followed from their index date until health plan disenrollment or the end of data availability (31 March 2010). People with insulin use during the baseline period or any time following the index date were excluded from the analysis. Selected people were classified as either having or not having evidence of medical care for a hypoglycaemic event during the study period, based on ICD-9-CM codes for hypoglycaemia (Table S6) at any place of service [22]. As in previous studies of hypoglycaemia, these observed hypoglycaemia events are considered as a proxy for greater underlying hypoglycaemia risk throughout the study period [5,23,24]. To achieve a computationally manageable study population, a random subsample of people without hypoglycaemia was chosen to achieve a 5 : 1 ratio to the sample with hypoglycaemia.

Baseline characteristics assessed before the index date included demographics, the Charlson comorbidity index (CCI) [25], common comorbidities of diabetes (obesity, mental disorders, neurological disorders, cardiovascular disorders, endocrine disorders and renal disorders) [4,26,27], health conditions associated with accident risk (epilepsy, stroke and substance abuse) [28], healthcare resource use [inpatient, outpatient and emergency room (ER) utilization] and type of index antidiabetes drug (see Table S6 for ICD-9-CM codes associated with each comorbidity). Baseline characteristics were compared between people with and without hypoglycaemia during the study period using chi-squared tests for categorical variables and Wilcoxon rank-sum tests for continuous variables. Accidents resulting in hospital visits during the study period were identified from inpatient and ER claims based on ICD-9-CM codes and were then grouped into three categories: accidental falls, motor vehicle accidents and other accidents (which included accidents occurring either at home, such as during housework or in relation to occupation, physical exertion, striking or being struck by an object, suffocation, foreign body entering eye/orifice, explosion and unspecified) (see Table S6 for ICD-9-CM codes).

For each accident outcome, a multivariable Cox proportional hazard model was used to assess the association between hypoglycaemia and occurrence of a first accident during the study period. Models were adjusted for baseline age 65 or older, gender, comorbidities of diabetes, conditions associated with accident risk, CCI, history of inpatient admission, index prescription for sulphonylurea and index prescription for thiazolidinedione. Adjusted hazard ratios (HRs) and corresponding 95% confidence intervals were estimated to compare people with, versus those without, a hypoglycaemia-related claim. Age group-specific HRs, for those aged ≥ or <65 years, were also estimated for the association between hypoglycaemia and each accident type by including an age-by-hypoglycaemia interaction term in the multivariable model. Annual incidence rates for accidents among people with, and without, hypoglycaemia were predicted based on the multivariable Cox model for hypothetical people with average baseline characteristics. A p-value of less than 0.05 was considered statistically significant.

As accidents could hypothetically increase the risk of hypoglycaemic events, especially if a person's eating routine is disrupted, a sensitivity analysis was conducted by excluding accidents that occurred within the 2 weeks before a hypoglycaemia claim. The time frame of 2 weeks was chosen for this sensitivity analysis based on the observation that three quarters of hypoglycaemia events among people with diabetes observing Ramadan occurred within the first 2 weeks of daytime fasting [29–31].

All analyses were conducted using SAS statistical software version 9.2 (SAS Institute Inc, Cary, NC, USA).

## Results

**Figure 1:**
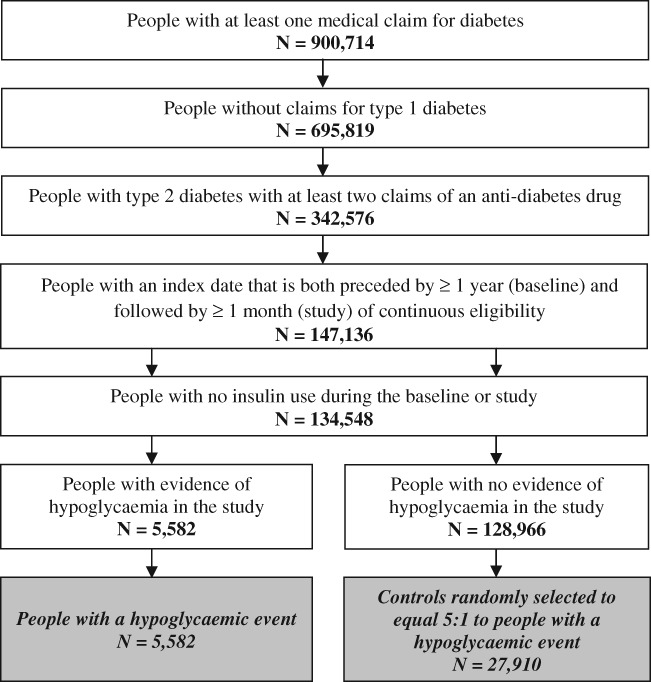
Sample selection flowchart.

A total of 1 47 136 people with type 2 diabetes initiated an antidiabetes drug and had continuous healthcare coverage for the 12-month baseline period. Of these, 1 34 548 (91.4%) did not use insulin during the baseline or study period. There were 5582 (4.1%) people with medical service claims related to hypoglycaemia and 1 28 966 without, of whom 27 910 were randomly sampled to serve as controls in a 1 : 5 ratio (Figure 1).

Compared with people without hypoglycaemia, those with hypoglycaemia had a higher average baseline CCI and were more likely to have baseline inpatient admissions or visits to hospital emergency departments, mental disorders, neurological disorders, endocrine disorders, renal disorders, epilepsy and stroke. People with hypoglycaemia were also more likely to have initiated a sulphonylurea as their index drug (Table 1).

**Table 1:** Characteristics of people with type 2 diabetes with, and without, hypoglycaemia

	Number (%)		
		
	Hypoglycaemia (n = 5582)	No hypoglycaemia (n = 27 910)	p Value
Male	2789 (50.0)	15 526 (55.6)	<0.001
Length of follow up, mean weeks (s.d.)	219 (124)	161 (116)	<0.001
Age, years, mean (s.d.)	60.40 (15.38)	59.50 (12.85)	<0.001
Age distribution (years)			<0.001
<18	71 (1.3)	65 (0.2)	
19–34	237 (4.2)	693 (2.5)	
35–44	503 (9.0)	2567 (9.2)	
45–54	1064 (19.1)	6215 (22.3)	
55–64	1472 (26.4)	8956 (32.1)	
65+	2235 (40.0)	9414 (33.7)	
Index prescription(s)			
Sulphonylureas	2131 (38.2)	7073 (25.3)	<0.001
Biguanides	3144 (56.3)	18 712 (67.0)	<0.001
α-glucosidase inhibitors	48 (0.9)	44 (0.2)	<0.001
Sitagliptin	57 (1.0)	634 (2.3)	<0.001
Incretin mimetics	27 (0.5)	175 (0.6)	0.21
Thiazolidinediones	829 (14.9)	4426 (15.9)	0.06
Medical resource use			
Inpatient (*N*, % with at least one)	1336 (23.9)	4544 (16.3)	<0.001
Hospital days, mean (s.d.)*	10.95 (30.74)	8.31 (14.61)	<0.001
Outpatient (*N*, % with at least one)	5180 (92.8)	25 877 (92.7)	0.83
Emergency room (*N*, % with at least one)	1650 (29.6)	6130 (22.0)	<0.001
Comorbidities of diabetes			
Obesity	254 (4.6)	1132 (4.1)	0.09
Mental disorders	847 (15.2)	3177 (11.4)	<0.001
Neurological disorders	962 (17.2)	2982 (10.7)	<0.001
Cardiovascular disorders	3370 (60.4)	16 455 (59.0)	0.05
Endocrine disorders	1965 (35.2)	11 992 (43.0)	<0.001
Renal disorders	919 (16.5)	3445 (12.3)	<0.001
Other conditions associated with accident risk			
Epilepsy	65 (1.2)	187 (0.7)	<0.001
Stroke	271 (4.9)	823 (2.9)	<0.001
Substance abuse	89 (1.6)	416 (1.5)	0.56
Charlson comorbidity index, mean (s.d.)	1.42 (1.70)	1.15 (1.38)	<0.001

Categorical variables are presented as number (%) and continuous variables are presented as mean (s.d.). P-values are calculated using the chi-squared test for categorical variables and Wilcoxon rank sum test for continuous variables.

* The mean number of hospital days was calculated conditional on having an inpatient stay in the baseline period.

A total of 1085 people had at least one accident resulting in a hospital visit during the study period: 308 of 5582 (5.5%) people with hypoglycaemia and 777 of 27 910 (2.8%) among those without. Falls were the most frequent type of accident. The distribution of hypoglycaemia claims and accidents is described further in Table S2. After adjusting for the potential confounders listed previously, medical claims for hypoglycaemia were associated with significantly greater hazards of any accident (adjusted HR: 1.39; CI: 1.21–1.59), accidental falls (adjusted HR: 1.36; CI: 1.13–1.65) and motor vehicle accidents (adjusted HR: 1.82; CI: 1.18–2.80). For every 10 000 patient-years of follow-up in people with medical claims for hypoglycaemia compared with those without, there was an expected 38 additional accidents of any type (137.4 vs. 99.2), 14 additional accidental falls (54.3 vs. 39.9) and 6.6 additional motor vehicle accidents (14.6 vs. 8.0) (Table 2). Further analyses of the category of ‘other’ accidents (related to housework or occupation, physical exertion and striking or being struck by object) are provided in Tables S4 and S5. Only 13 people experienced an accident within the 2 weeks before a hypoglycaemic event. Excluding these accidents did not alter the study findings (Table S1).

**Table 2:** Hypoglycaemia and risk of accidents

	Number (%)			Predicted incidence rate per 10 000 person-years (95% CI)	
			
Type of accident	Hypoglycaemia (n = 5582)	No hypoglycaemia (n = 27 910)	Hazard ratio (95% CI)	Hypoglycaemia	No hypoglycaemia
Any accident	308 (5.5)	777 (2.8)	1.39 (1.21, 1.59)*	137.4 (116.9, 157.8)	99.2 (88.2, 110.2)
Accidental fall	161 (2.9)	369 (1.3)	1.36 (1.13, 1.65)*	54.3 (42.3, 66.2)	39.9 (33.2, 46.6)
Motor vehicle accident	32 (0.6)	65 (0.2)	1.82 (1.18, 2.80)*	14.6 (7.6, 21.5)	8.0 (5.0, 11.0)
Other accident	129 (2.3)	361 (1.3)	1.33 (1.09, 1.64)*	57.0 (44.0, 70.0)	42.8 (35.6, 50.0)

Analyses were performed using multivariable Cox proportional hazard models which assessed the association between hypoglycaemia and occurrence of a first accident following initiation of an antidiabetes drug. Hazard ratio estimates were adjusted for demographics, baseline comorbidities, CCI and baseline resource use. Accidents refer to the first accident in the underlying category a person experienced. Therefore number of accidents from each subcategory may add to a number greater than those with ‘any accident’. CI, confidence interval.

* P-values < 0.05.

In the age-stratified analyses, people older and younger than 65 years had similar rates of any accident, and comparably increased hazards of any accident associated with hypoglycaemia (adjusted HR: 1.35 and 1.46, respectively; Table 3). However, the risk of falls was twice as high among the older people compared with younger individuals, and, among those who were older, hypoglycaemia was significantly associated with a greater than 50% increase in the hazard of falls (adjusted HR: 1.52; CI: 1.18–1.95). Hypoglycaemia was not associated with a statistically significant increase in the hazard of falls among younger people with diabetes (Table 3). Younger people had an overall greater risk of motor vehicle accidents compared with older people. Among the younger people, hypoglycaemia was significantly associated with a greater than 130% increase in the risk of motor vehicle accidents (adjusted HR: 2.31; CI: 1.44–3.70). Hypoglycaemia was not associated with motor vehicle accident risk among the older individuals (Table 3). Although HRs for hypoglycaemia differed numerically between older and younger people, the age-by-hypoglycaemia interaction was not statistically significant (Table S3).

**Table 3:** Hypoglycaemia and risk of accidents stratified by age group

	Number (%)			Predicted incidence rate per 10 000 person-years (95% CI)	
			
Population with type 2 diabetes and nature of accident	Hypoglycaemia	No hypoglycaemia	Hazard ratio (95% CI)	Hypoglycaemia	No hypoglycaemia
Age <65 years	(n = 3347)	(n = 18 496)			
Any accident	175 (5.2)	507 (2.7)	1.35 (1.14, 1.61)*	138.5 (114.1, 162.9)	102.8 (90.3, 115.3)
Accidental fall	62 (1.9)	193 (1.0)	1.17 (0.88, 1.57)	42.7 (30.4, 55.1)	36.4 (29.5, 43.4)
Motor vehicle accident	28 (0.8)	49 (0.3)	2.31 (1.44, 3.70)*	23.9 (12.4, 35.5)	10.4 (6.3, 14.4)
Other accident	96 (2.9)	275 (1.5)	1.43 (1.13, 1.81)*	76.0 (57.2, 94.7)	53.1 (43.7, 62.5)
Age ≥65 years	(n = 2235)	(n = 9414)			
Any accident	133 (6.0)	270 (2.9)	1.46 (1.18, 1.80)*	135.5 (108.0, 162.9)	92.9 (78.9, 106.9)
Accidental fall	99 (4.4)	176 (1.9)	1.52 (1.18, 1.95)*	78.9 (58.3, 99.4)	52.0 (41.4, 62.6)
Motor vehicle accident	4 (0.2)	16 (0.2)	0.79 (0.26, 2.38)	4.7 (0.0, 9.7)	6.0 (2.6, 9.4)
Other accident	33 (1.5)	86 (0.9)	1.23 (0.82, 1.85)	38.0 (23.6, 52.3)	30.8 (23.1, 38.6)

Analyses were performed using multivariable Cox proportional hazard models which assessed the association between hypoglycaemia and occurrence of a first accident following initiation of an antidiabetes drug. Hazard ratio estimates were adjusted for demographics, baseline comorbidities, CCI and baseline resource use. Interactions between age stratum and hypoglycaemia [hypoglycaemia and under age 65 (top panel) and hypoglycaemia and age 65 or older (bottom panel)] were included in the models. Accidents refer to the first accident in the underlying category a person experienced. Therefore number of accidents from each subcategory may add to a number greater than those with ‘any accident’. CI, confidence interval.

* P-values < 0.05.

## Discussion

This study has retrospectively evaluated associations between hypoglycaemia and accident risk among people receiving antidiabetes drugs (without insulin) in a large American claims database. In this population, people with medical claims related to hypoglycaemia had significantly greater accident risks than those without. Associations differed numerically by age group. In people younger than 65 years, hypoglycaemia was associated with a greater risk of motor vehicle accidents; in older people, hypoglycaemia was associated with a greater risk of accidental falls. These findings extend the literature linking hypoglycaemia to accident risk beyond people with insulin-treated diabetes.

Associations between accident risk and hypoglycaemia identified in this study are similar in magnitude to associations with other known accident risk factors. Risk factors for falls have been widely studied. In a nursing home setting, dementia has been associated with a 74% greater risk of falls [32]. Among elderly hospital inpatients, hearing impairment has been associated with a 49% greater risk of falls [33]. Lower HbA1c has been numerically associated with a higher risk of falls in a previous study of well-functioning older adults using oral hypoglycaemic medications but not insulin [15]. Among people with type 2 diabetes receiving oral antidiabetes drugs under employer-sponsored Medicare supplemental plans, not excluding those using insulin, hypoglycaemia has been associated with increased risk of fall-related fractures [16]. Recognized health-related risk factors for motor vehicle accidents include heart disease, stroke and peripheral vision score ≤75%, which have been associated with 40–80% greater risks of motor vehicle accidents in drivers aged 65 or older [21]. These risk factors can also affect driving licensure. A previous study of 122 people taking antidiabetes medication without insulin estimated that, on average, three hypoglycaemia-induced traffic accidents occur per 10 million km driven [20].

Although hypoglycaemia was associated with an increased overall accident risk both in older and younger people, associations with specific accident types appeared to differ between younger and older people. Stronger evidence associating hypoglycaemia with greater motor vehicle accident risk was observed among people who were aged less than 65. Stronger evidence associating hypoglycaemia with a greater risk of falls was observed among older people. For each accident type, the age group with stronger evidence for an association with hypoglycaemia also had a greater absolute risk without hypoglycaemia. The absence of strong evidence for an association between hypoglycaemia and motor vehicle accidents in the older subgroup is not surprising. Previous research in the UK has documented that the automobile accident risk is lower among older drivers with insulin-treated diabetes compared with non-diabetic aged-matched controls. This was considered to be associated with awareness of declining physical capabilities resulting in possible modifications of driving behaviour. For example, out of caution, elderly people with diabetes may restrict their driving to daytime hours, drive shorter distances and refrain from driving in inclement weather compared with those who do not have diabetes [34].

This study has a number of limitations. First, only moderate to severe hypoglycaemia events are likely to result in medical service use that is detectable via insurance claims. People who experienced hypoglycaemia but did not receive medical services related to hypoglycaemia would contribute to the hypoglycaemia-free group in this study. Such misclassification would bias the estimated associations with accident risk towards the null, making the present estimates conservative. Given that the prevalence of mild hypoglycaemia is generally much higher than episodes of severe hypoglycaemia in people with type 2 diabetes (39 vs. 7% in one study [17]), further study of the impact of mild hypoglycaemia in this population is warranted. Secondly, it should be emphasized that this study regards observed hypoglycaemia events as a proxy for greater underlying hypoglycaemia risk throughout the study period. It was not possible to directly assess blood glucose values in this large claims database. Thirdly, as in any observational study, it is possible that unidentified or unmeasured factors are confounding the observed associations between hypoglycaemia and accident risk [35]. In particular, laboratory values, such as HbA1c, and patient characteristics, such as body mass index, duration of diabetes or alcohol use, are not available in the claims data. In addition, claims data may contain errors in coding or missing data, although these should affect each study group in equal measure.

## Conclusions

Hypoglycaemia-associated accidents are a well-appreciated concern for people receiving insulin therapy. This study has shown that hypoglycaemia is also associated with increased accident risks in people with type 2 diabetes receiving antidiabetes drugs without insulin. It is important that physicians are aware of this association and educate people with diabetes about the warning symptoms of hypoglycaemia and the precautions that can be taken to avoid motor vehicle accidents and falls. Hypoglycaemia and accident risk may also be considered when considering treatment options, especially for add-on or follow-on therapies for people with an inadequate response to, or intolerance of, metformin monotherapy [36]. Sulphonylureas significantly increase hypoglycaemia risk [17,18], and this increased risk is augmented when thiazolidinediones are added to sulphonylurea therapy [37]. However, clinical trial results for dipeptidyl-peptidase 4 inhibitors have not shown an association between treatment and hypoglycaemia [38,39]. Further study of risk factors for hypoglycaemia in non-insulin-treated diabetes is required to develop procedures and policies to limit hypoglycaemia and accident risk in this population [40].

## Supporting Information

Table S1. Sensitivity analysis of association between hypoglycaemia and risk of accidents.

Table S2. Frequency of hypoglycaemic episodes by accident status.

Table S3. Interaction between age, accident risk and hypoglycaemia.

Table S4. Hypoglycaemia and risk of other accidents (i.e. those caused by housework or occupation, physical exertion or by striking or being struck by an object).

Table S5. Analyses of hypoglycaemia and risk of other accidents with interactions between age stratum and hypoglycaemia (i.e. those caused by housework or occupation, overexertion, or by striking or being struck by an object).

Table S6. ICD-9 codes used in the analysis.
