# Supplementary material for: Hypoglycaemia and accident risk in people with type 2 diabetes mellitus treated with non-insulin antidiabetes drugs
Source: Diabetes Obes Metab. 2012 Nov 22;15(4):335–41. doi: 10.1111/dom.12031 (PMC3593162; doi:10.1111/dom.12031)
Supplement: Supplementary file 1 [file dom0015-0335-SD1.doc]

Table S1. Sensitivity analysis of association between hypoglycaemia and risk of accidents.

|  | **No. (%)** | | | | | |  |  | **Predicted Incidence Rate**  **per 10 000 Person-years (95% CI)** | | | |
| --- | --- | --- | --- | --- | --- | --- | --- | --- | --- | --- | --- | --- |
|  | **Hypoglycaemia**  **(n = 5,582)** | | **No hypoglycaemia**  **(n = 27,910)** | | | | **Hazard Ratio (95% CI)** | |  |  |  |  |
|  |  |  |  |  |  |  |  |  | **Hypoglycaemia** | **No hypoglycaemia** | | |
| Any accident | 298 | (5.3) | | 777 | (2.8) | 1.34 (1.17-1.54) | | | 133.2 (113.2, 153.3) | | 99.3 (88.3, 110.4) |  |
| Accidental fall | 154 | (2.8) | | 369 | (1.3) | 1.31 (1.08-1.59) | | | 52.3 (40.6, 64.0) | | 40.0 (33.2, 46.8) |  |
| Motor vehicle accident | 31 | (0.6) | | 65 | (0.2) | 1.75 (1.13-2.71) | | | 14.1 (7.3, 20.9) | | 8.1 (5.0, 11.1) |  |
| Other accident | 126 | (2.3) | | 361 | (1.3) | 1.30 (1.06-1.60) | | | 55.7 (42.9, 68.6) | | 42.9 (35.6, 50.1) |  |

Abbreviation: CI, confidence interval.

Notes: 1. Analyses were performed using multivariable Cox proportional hazard models which assessed the association between hypoglycaemia and occurrence of a first accident following initiation of an anti-diabetes drug. 2. Hazard ratio estimates were adjusted for demographics, baseline comorbidities, CCI and baseline resource use. 3. Accidents that occurred less than 2 weeks before a claim for a hypoglycaemic event were excluded. 4. Accidents refer to the first accident in the underlying category a person experienced. Therefore number of accidents from each subcategory may add to a number greater than those with “any accident.”
