# Supplementary material for: Hypoglycaemia and accident risk in people with type 2 diabetes mellitus treated with non-insulin antidiabetes drugs
Source: Diabetes Obes Metab. 2012 Nov 22;15(4):335–41. doi: 10.1111/dom.12031 (PMC3593162; doi:10.1111/dom.12031)
Supplement: Supplementary file 2 [file dom0015-0335-SD2.doc]

Table S2. Frequency of hypoglycaemic episodes by accident status.

| **Number of hypoglycaemic episodes** | | **Accident (n = 1,085)** | | | **No Accident (n = 32,407)** | |
| --- | --- | --- | --- | --- | --- | --- |
| 0 | 777 | | (71.6%) | 27,133 | | (83.7%) |
| 1 | 209 | | (19.3%) | 3,493 | | (10.8%) |
| 2 | 59 | | (5.4%) | 865 | | (2.7%) |
| 3 or more | 40 | | (3.7%) | 916 | | (2.8%) |

Notes: 1. All data are presented as No. (%).
