# Supplementary material for: Hypoglycaemia and accident risk in people with type 2 diabetes mellitus treated with non-insulin antidiabetes drugs
Source: Diabetes Obes Metab. 2012 Nov 22;15(4):335–41. doi: 10.1111/dom.12031 (PMC3593162; doi:10.1111/dom.12031)
Supplement: Supplementary file 3 [file dom0015-0335-SD3.doc]

Table S3. Interaction between age, accident risk and hypoglycaemia.

|  | **65 or Older among those with no hypo** |  | **Had a Hypoglycaemic event among those younger than 65** |  | **Had a Hypoglycaemic event and 65 or Older** |
| --- | --- | --- | --- | --- | --- |
|  | Hazard Ratio |  | Hazard Ratio |  | Hazard Ratio |
| Any accident | 0.90 |  | 1.35* |  | 1.08 |
| Accidental fall | 1.43* |  | 1.17 |  | 1.29 |
| Motor vehicle accident | 0.58 |  | 2.31* |  | 0.34 |
| Other accident | 0.58* |  | 1.43* |  | 0.86 |

Notes: 1. Analyses were performed using multivariable Cox proportional hazard models which assessed the association between hypoglycaemia and occurrence of a first accident following initiation of an anti-diabetes drug. 2. Hazard ratio estimates were adjusted for demographics, baseline comorbidities, CCI and baseline resource use. 3. To account for the possibility of age having a differential effect on the relationship between hypoglycaemia and accidents, the interaction term has been included between hypoglycaemia and aged 65 or older. 5. Asterisks represent p values <0.05.
