# Supplementary material for: Hypoglycaemia and accident risk in people with type 2 diabetes mellitus treated with non-insulin antidiabetes drugs
Source: Diabetes Obes Metab. 2012 Nov 22;15(4):335–41. doi: 10.1111/dom.12031 (PMC3593162; doi:10.1111/dom.12031)
Supplement: Supplementary file 4 [file dom0015-0335-SD4.doc]

Table S4. Hypoglycaemia and risk of other accidents (i.e. those caused by housework or occupation, physical exertion, or by striking or being struck by an object).

|  | **No. (%)** | | | |  | |  | **Predicted Incidence Rate** | | | |
| --- | --- | --- | --- | --- | --- | --- | --- | --- | --- | --- | --- |
|  | **Hypoglycaemia** | | **No hypoglycaemia** | | **Hazard Ratio (95% CI)** | | | **per 10,000 Person-years (95% CI)** | | | |
|  | **(n = 5,582)** | | **(n = 27,910)** | |  |  |  | **Hypoglycaemia** | | **No hypoglycaemia** | |
| Accident caused by housework or occupation | 36 | (0.6) | 110 | (0.4) | 1.29 | (0.88-1.89) | | 14.1 | (7.9, 20.3) | 11.0 | (7.3, 14.6) |
| Accident caused by overexertion | 42 | (0.8) | 95 | (0.3) | 1.62 | (1.12-2.35) | | 16.6 | (9.6, 23.6) | 10.3 | (6.9, 13.7) |
| Accident caused by striking or being struck by object | 31 | (0.6) | 77 | (0.3) | 1.44 | (0.94-2.21) | | 13.2 | (6.9, 19.5) | 9.2 | (5.9, 12.4) |

Abbreviation: CI, confidence interval.

Notes: 1. Analyses were performed using multivariable Cox proportional hazard models which assessed the association between hypoglycaemia and occurrence of a first accident following initiation of an anti-diabetes drug. 2. Hazard ratio estimates were adjusted for demographics, baseline comorbidities, CCI and baseline resource use. 3. Accidents refer to the first accident in the underlying category a person experienced. Therefore number of accidents from each subcategory may add to a number greater than those with “any accident.”
