# Supplementary material for: Hypoglycaemia and accident risk in people with type 2 diabetes mellitus treated with non-insulin antidiabetes drugs
Source: Diabetes Obes Metab. 2012 Nov 22;15(4):335–41. doi: 10.1111/dom.12031 (PMC3593162; doi:10.1111/dom.12031)
Supplement: Supplementary file 5 [file dom0015-0335-SD5.doc]

Table S5. Analyses of hypoglycaemia and risk of other accidents with interactions between age stratum and hypoglycaemia (i.e. those caused by housework or occupation, overexertion, or by striking or being struck by an object).

|  | | | | **No. (%)** | | | | **Hazard Ratio (95% CI)** | | **Predicted Incidence Rate**  **per 10,000 Person-years (95% CI)** | | | | |  |
| --- | --- | --- | --- | --- | --- | --- | --- | --- | --- | --- | --- | --- | --- | --- | --- |
|  | | | | **Hypoglycaemia** | | **No hypoglycaemia** | |  |  |  |  |  |  |  |  |
|  |  |  |  |  |  |  |  |  |  | **Hypoglycaemia** | | **No hypoglycaemia** | | |  |
| **Age < 65 years** |  |  |  | **(n = 3,347)** | | **(n = 18,496)** | |  | |  | | | | | |
| Accident caused by housework or occupation | | | | 26 | (0.8) | 84 | (0.5) | 1.34 | (0.86-2.09) | 18.3 | (9.6, 27.1) | 13.7 | (9.0, 18.4) | |  |
| Accident caused by overexertion | | | | 37 | (1.1) | 76 | (0.4) | 1.94 | (1.30-2.89) | 27.3 | (15.6, 39.0) | 14.1 | (9.3, 18.8) | |  |
| Accident caused by striking or being struck by object | | | | 24 | (0.7) | 62 | (0.3) | 1.54 | (0.96-2.49) | 19.5 | (9.7, 29.2) | 12.6 | (8.0, 17.2) | |  |
| **Age ≥ 65 years** | |  |  | **(n = 2,235)** | | **(n = 9,414)** | |  |  |  | | | | | |
| Accident caused by housework or occupation | | | | 10 | (0.4) | 26 | (0.3) | 1.24 | (0.60-2.60) | 9.4 | (2.8, 16.0) | 7.6 | | (4.0, 11.2) | |
| Accident caused by overexertion | | | | 5 | (0.2) | 19 | (0.2) | 0.87 | (0.32-2.35) | 5.9 | (0.4, 11.4) | 6.8 | | (3.3, 10.3) | |
| Accident caused by striking or being struck by object | | | | 7 | (0.3) | 15 | (0.2) | 1.48 | (0.60-3.65) | 8.5 | (1.6, 15.5) | 5.8 | | (2.4, 9.1) | |

Abbreviation: CI, confidence interval.

Notes: 1. Analyses were performed using multivariable Cox proportional hazard models which assessed the association between hypoglycaemia and occurrence of a first accident following initiation of an anti-diabetes drug. 2. Hazard ratio estimates were adjusted for demographics, baseline comorbidities, CCI and baseline resource use. 3. Accidents refer to the first accident in the underlying category a person experienced. Therefore number of accidents from each subcategory may add to a number greater than those with “any accident.”
