# Supplementary material for: Hypoglycaemia and accident risk in people with type 2 diabetes mellitus treated with non-insulin antidiabetes drugs
Source: Diabetes Obes Metab. 2012 Nov 22;15(4):335–41. doi: 10.1111/dom.12031 (PMC3593162; doi:10.1111/dom.12031)
Supplement: Supplementary file 6 [file dom0015-0335-SD6.doc]

Table S6. ICD-9 codes used in the analysis.

| ICD-9 codes for hypoglycaemia |
| --- |
| - - - Included   - 250.8 (Diabetes with other specified manifestations, which is specific for diabetic hypoglycaemia or hypoglycaemic shock without concurrent coding for ulceration (707.10-707.9) or diabetic bone changes (731.8)),   - 251.0 (Hypoglycaemic coma),   - 251.1(Other specified hypoglycaemia),   - 251.2 (Hypoglycaemia unspecified), and   - 250.3 (Diabetes with other coma).     - Excluded   - 270.3 (leucine-induced hypoglycaemia),   - 775.6 (neonatal hypoglycaemia), and   - 775.0 (hypoglycaemia in an infant born to a diabetic mother). |
| ICD-9 codes for accidents |
| - - - Accidental falls (E880.1, E880.9, E881.0, E883.9, E884.2, E884.6, E884.9, E885, E886.0, E887, E888 and E917.7),     - Motor vehicle accidents (E810-E819, E8210, E8219 and E825.0),     - Accidents caused by striking or being struck by object (E824.9, E886.9, E916, E917.0, E917.4, E917.9 and E918),     - Other accidents such as those caused by housework/occupation, overexertion, striking or being struck by an object, suffocation, foreign body entering eye/orifice, explosion, or unspecified (E919.0, E919.8, E920, E924.0, E924.1, E924.9, E926.2, E927, E824.9, E886.9, E916, E917.0, E917.4, E917.9, E918, E912, E914, E915, E923.8, E928.8 and E928.9)     - Any accident (all codes specified above) |
| ICD-9 codes for comorbidities |
| - - - Obesity (278)     - Mental Disorders (290 – 319)     - Neurological Disorders (250.6, 337.1, 354, 355, 357.2, 358.1, 356.8, 430-432, 433, 434, 435, 436, 437, 438, 713.5, 729.2, 707, 730)     - Cardiovascular Disorders (250.7, 398.91, 401-405, 410-414, 425, 426-427, 428, 429.1, 429.2, 429.3, 440-448, 451, 452, 453, 454, 458, 459, 707, 785.4, 885-887, 895-897)     - Endocrine Disorders (259.4, 259.8, 261, 271.0, 271.1, 271.4, 272.0-272.4, 272.7, 273.3, 275.0, 276.7)     - Renal Disorders (250.4, 271.4, 580-588, 590, 593, 595, 596, 599, 791.0, 791.5, V420, V451, V56) |
| ICD-9 codes for conditions associated with higher accident rates |
| - - - Epilepsy (345, 780.39)     - Stroke (433.01, 433.11, 433.21, 433.31, 433.81, 433.91, 434.01, 434.11, 434.91, 435.9, 436, 437.1, 437.9)     - Substance abuse (303 – 305) |
